# Chemical Profile and Related Antioxidant and Anti-Inflammatory Activities of Leaf Essential Oil from *Aspilia rudis* Oliv. & Hiern

**DOI:** 10.3390/plants15060865

**Published:** 2026-03-11

**Authors:** Didjour Albert Kambiré, Guy Blanchard Boué, Mathieu Paoli, Ange Bighelli, Jean Brice Boti, Zanahi Félix Tonzibo, Félix Tomi

**Affiliations:** 1 UPR de Chimie, Département de Mathématiques, Physique et Chimie, UFR des Sciences Biologiques, Université Peleforo Gon Coulibaly, Korhogo BP 1328, Côte d’Ivoire; dakambire@gmail.com; 2 UFR des Sciences et Technologies, Université Alassane Ouattara, Bouaké BP V18 01, Côte d’Ivoire; blanchardboue@uao.edu.ci; 3 Laboratoire Sciences Pour l’Environnement, Université de Corse—CNRS, UMR 6134 SPE, Route des Sanguinaires, 20000 Ajaccio, France; paoli_m@univ-corse.fr (M.P.); bighelli_a@univ-corse.fr (A.B.); 4 Laboratoire de Constitution et Réaction de la Matière, UFR-SSMT, Université Félix Houphouët-Boigny, Abidjan BP V34, Côte d’Ivoire; jeanbriceboti@hotmail.fr (J.B.B.); tonzibz@yahoo.fr (Z.F.T.)

**Keywords:** *Aspilia rudis*, leaf oil, chemical composition, antioxidant, anti-inflammatory

## Abstract

The present study reports, for the first time, the chemical composition of the leaf essential oil (LEO) from *Aspilia rudis* as well as its antioxidant and anti-inflammatory activities. Analysis combining GC(RI), GC-MS and ^13^C-NMR of 36 samples identified 58 compounds representing 96.7–99.3% of the whole composition. Statistical analyses revealed chemical variability in three clusters, each composed of samples from the same sampling site. Cluster I is dominated by germacrene D (27.2 ± 2.7%), α-pinene (24.0 ± 2.9%) and (*E*)-β-caryophyllene (13.1 ± 1.7%), cluster II by α-pinene (38.9 ± 2.4%) and germacrene D (19.1 ± 3.6%), while the prevalent compound of cluster III is α-pinene (51.9 ± 5.3%), followed by β-pinene (11.7 ± 1.7%) and germacrene D (10.7 ± 2.2%). The oil samples S10, S26 and S36 demonstrated antioxidant activity (DPPH: EC_50_ = 43.8 ± 1.0, 28.5 ± 1.0 and 38.8 ± 1.0 µg/mL, respectively; ABTS: TEAC = 17.16 ± 0.70, 23.35 ± 1.32 and 18.76 ± 0.64 µmol TE/mg EO, respectively) and anti-inflammatory activity through the LOX inhibition assay (IC_50_ = 34.9 ± 1.0, 32.1 ± 1.0 and 22.2 ± 1.0 µg/mL, respectively). The activities of *A. rudis* LEO may be related to its main compounds and thymol, all known for their various pharmacological and biological properties, and probably to synergistic effects.

## 1. Introduction

Over the years, essential oils have been used for various purposes and are the subject of countless studies for applications as bioactive agents. The natural volatile compounds of essential oils have demonstrated numerous biological activities such as anticancer, antimicrobial, antiviral, insecticidal, anti-inflammatory, antiprotozoal, neuroprotective, and antioxidant properties [1]. Given the wide range of applications of essential oils, including in perfumery, cosmetics, aromatherapy, drug discovery and food industries, it appears necessary to characterize them chemically in order to assess their specificity and control their quality [2].

The genus *Aspilia* Thouars belongs to the Asteraceae family and comprises approximately 60 species, 21 of which are found in tropical Africa and Madagascar. *Aspilia rudis* Oliv. & Hiern. (synonym of *Wedelia rudis* (Baker) H. Rob.) is a robust, often perennial herb that can reach 0.9 to 1.2 m in height. It has an erect stem with short-petioled leaves measuring 3 to 13 cm long and 0.8 to 3 cm wide. The leaf apex is acuminate, the upper surface is scabious (rough), and the lower surface is hispid (covered with stiff hairs). The pale-yellow inflorescences are arranged in small, dense terminal cymes. This species is only found in tropical Africa and primarily in wooded savanna and forest areas [3]. *A. rudis* is traditionally used to treat various ailments, including skin rashes, digestive disorders, stomach pain, diarrhea and to heal wounds and burns. In Côte d’Ivoire, this species is used in steam baths to treat dracunculiasis caused by the Guinea worm. This plant also possesses anti-inflammatory and antitussive properties. In Central and Southern Africa, its leaves are swallowed whole by chimpanzees to treat parasitic infections [4,5].

Two species of the genus *Aspilia* have been investigated for their essential oil composition. The LEO of *Aspilia africana* (Pers.) C.D. Adams from Nigeria is dominated either by germacrene D (15.6–28.4%), α-pinene (13.6–17,1%) and β-caryophyllene (7,5–10.8%) [6,7], or by α-cubebene (31.1%), followed by α-copaene (7.0%) and α-pinene (6.7%) [8]. In Cameroon, limonene (23.3%), α-pinene (21.8%), germacrene D (6.7%) and β-caryophyllene (5.8%) are the main constituents of the same plant leaf oil [9]. The LEO of *Aspilia helianthoides* (Schumach. & Thonn.) Oliv. & Hiern is dominated by α-pinene (26.5–38.7%), germacrene D (13.8–24.4%), limonene (7.5–9.4%) and β-pinene (6.7–7.4%) or by germacrene D (45.0–54.2%), β-caryophyllene (8.7–13.2%), and δ-cadinene (7.6–8.2%) [9].

This study is a continuation of our work on chemically characterizing essential oils, extracted from aromatic and medicinal plants of Côte d’Ivoire [10]. To our knowledge, no previous studies have been conducted on the chemical constituents and biological activities of *A. rudis*. Therefore, the present work aims to describe, for the first time, the chemical profile of the leaf essential oil of the Ivorian species, and to evaluate its antioxidant and anti-inflammatory activities.

## 2. Results and Discussion

### 2.1. Chemical Profile of the Leaf Essential Oil

The chemical composition of the LEO of *A. rudis* has been investigated for the first time. Thirty-six samples of fresh leaves were harvested in three locations of Southern Côte d’Ivoire, including the edge of the Yapo-Abbé Forest, Cocody, and nearby Agou. Essential oils were extracted by hydrodistillation, and the yields calculated on a weight (*w*/*w*) basis varied from 0.126 to 0.224% (Supplementary Materials Table S1). The analyses of the samples were carried out through a combination of GC(RI) (on non-polar and polar capillary columns), GC-MS (on a non-polar capillary column) and ^13^C-NMR, using a computerized method. This method developed at the University of Corsica allows for the identification of components that are compiled in the laboratory-made ^13^C-NMR spectral data library and present at a content greater than 0.4–0.5% [11].

The three analytical techniques mentioned above led to the identification of 58 constituents, accounting for 96.7–99.3% of the whole chemical composition (Supplementary Materials Table S2). As germacrene B, a thermolabile compound, and γ-elemene, its corresponding rearrangement product, were both detected by GC-MS and ^13^C-NMR, their correct contents were assessed through a combination of GC(FID) and ^13^C-NMR [11]. The LEO of *A. rudis* is very rich in monoterpene hydrocarbons (27.7–80.4%) and sesquiterpene hydrocarbons (15.5–56.8%). The identified predominant compounds were α-pinene (19.6–59.8%), germacrene D (7.3–30.8%), (*E*)-β-caryophyllene (2.1–16.8%) and β-pinene (3.9–13.5%). Thymol (up to 7.8%), α-humulene (up to 7.1%) and limonene (up to 5.0%) were also detected at noticeable contents in some samples. The main constituent contents varied from sample to sample; statistical analyses were performed on the 36 samples’ compositions in order to evaluate their chemical variability. Only compounds present at a concentration greater than or equal to 1% in at least one sample were taken into account.

### 2.2. Chemical Variability in the Leaf Essential Oil

**Figure 1:**
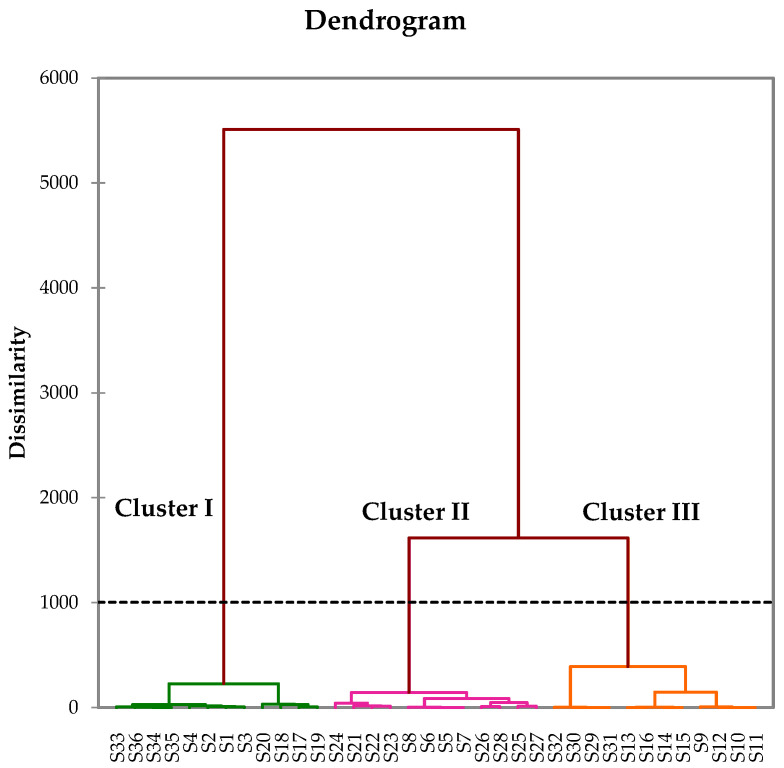
Hierarchical cluster analysis of the 36 LEO samples from *A. rudis.* Cluster I: green; cluster II: magenta; cluster III: orange.

**Figure 2:**
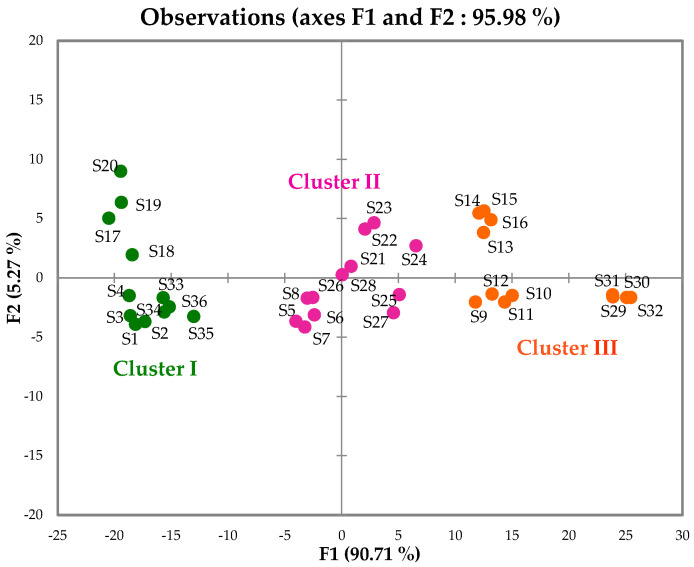
PCA of the 36 LEO samples from *A. rudis.* Cluster I: green; cluster II: magenta; cluster III: orange.

The 36 samples’ compositions were subjected to hierarchical clustering (HC) and principal component analysis (PCA). The Kaiser–Meyer–Olkin criterion value was 0.998, confirming the suitability of the complete correlation matrix (Supplementary Materials Table S3). The dendrogram of the HC exhibited three clusters, each corresponding to a sampling site (Figure 1): cluster I (12 samples from the edge of the Yapo-Abbé Forest), cluster II (12 samples from Agou) and cluster III (12 samples from Cocody). The samples were collected across rainy (November 2024) and dry (January and March 2025) seasons. Clustering aligned primarily with collection sites and seasonality cannot be robustly excluded without dedicated testing (Table S1). For the PCA, the principal factors F1 (90.71%) and F2 (5.27%) were selected according to the scree plot (Supplementary Materials Figure S1 and Table S4). They accounted for 95.98% of the total variance of the chemical composition (Figure 2). Although all samples are distributed along the main axis F1 (variable contributions: 66.1% for α-pinene; 22.4% for germacrene D; 6.9% for (*E*)-β-caryophyllene; and 3.0% for β-pinene), the three clusters remain observable. This suggests quantitative variability of the major constituents of the different clusters. The mean content (M) and the standard deviation (SD) of the main compounds that discriminated the clusters are reported in Table 1.

Cluster I (samples from the edge of the Yapo-Abbé Forest) is dominated by germacrene D (27.2 ± 2.7%), α-pinene (24.0 ± 2.9%) and (*E*)-β-caryophyllene (13.1 ± 1.7%), followed by β-pinene (5.8 ± 1.3%) and α-humulene (5.2 ± 1.1%). The main constituents of cluster II (samples from Agou) are α-pinene (38.9 ± 2.4%) and germacrene D (19.1 ± 3.5%), followed by β-pinene (9.3 ± 1.9%) and (*E*)-β-caryophyllene (8.4 ± 1.6). Cluster II differs from cluster I by having higher contents of α- and β-pinene, and lower contents of germacrene D and (*E*)-β-caryophyllene. Thus, cluster I is richer in sesquiterpene hydrocarbons (54.3 ± 2.3%) than cluster II (36.5 ± 5.2%), which has higher amount of monoterpene hydrocarbons (56.8 ± 4.8% vs. 36.1 ± 4.6% for Cluster I). Similar to cluster II, cluster III (samples from Cocody) is also dominated by monoterpene hydrocarbons (72.2 ± 5.6%). The prevalent compound in this cluster is α-pinene (51.9 ± 5.3%), followed by β-pinene (11.7 ± 1.7%) and germacrene D (10.7 ± 2.2%). The chemical compositions of samples S10, S26 and S36, which are representative of clusters III, II and I, respectively, are compiled in Table 2. Although the chemical composition of the LEO of *A. rudis* appears similar to that of *A. africana* and *A. helianthoides*, two other species from the genus *Aspilia*, some noticeable differences are observable. Indeed, as already mentioned, the major compounds in the leaf oil of *A. africana* from Nigeria are germacrene D (15.6–28.4%), α-pinene (13.6–17.1%) and β-caryophyllene (7.5–10.8%) [6,7], or α-cubebene (31.1%), followed by α-copaene (7.0%) and α-pinene (6.7%) [8]. The Cameroonian species is dominated by limonene (23.3%), α-pinene (21.8%), germacrene D (6.7%) and β-caryophyllene (5.8%) [9]. In the present study, α-cubebene was not detected in the LEO of *A. rudis*, while limonene is present at a relatively low content (0.8–5.0%). Conversely, thymol (up to 7.8% in this study) was not detected in previous studies on *A. africana* and *A. helianthoides*. The latter exhibits two chemical compositions, dominated either by α-pinene (26.5–38.7%) and germacrene D (13.8–24.4%) or by germacrene D (45.0–54.2%) and β-caryophyllene (8.7–13.2%) [9]. Germacrene D, α-pinene and (*E*)-β-caryophyllene are the common major compounds of the three species (*A. rudis*, *A. africana* and *A. helianthoides*) and could be chemotaxonomic markers of the genus *Aspilia*.

### 2.3. Antioxidant and Anti-Inflammatory Activity of the LEO Samples from A. rudis

The LEO samples S10, S26 and S36, which are representative of clusters III, II and I, respectively, were used in the antioxidant and anti-inflammatory assays. These three samples exhibited chemical compositions close to the means of the clusters (Table 1). For each activity, technical replications were used (three hydro-distillations of the same vegetal material).

#### 2.3.1. DPPH• Radical Scavenging

The inhibition percentages and the effective concentrations required to reduce DPPH• radicals by 50% (EC_50_) were determined from regression lines representing the inhibition as a function of concentration (Supplementary Materials Figure S2). For a given sample, the lower the EC_50_ value, the higher its antioxidant capacity. The EC_50_ values were 43.8 ± 1.0, 28.5 ± 1.0, 38.8 ± 1.0, and 15.8 ± 1.0 µg/mL for samples S10, S26, S36, and vitamin C, respectively (Table 3). Statistical tests based on technical replicates indicated differences between these means (*p* < 0.05). All the tested LEO samples demonstrated DPPH• radical scavenging capacities, although they were lower than that of vitamin C, which was used as the positive control. Sample S26 exhibited the strongest antioxidant potential, followed by samples S10 and S36, which had with similar activities.

#### 2.3.2. ABTS+• Cation Radical Scavenging

The effective concentrations required to scavenge 50% of ABTS+• cation radicals (EC_50_) were determined from the linear regression of inhibition percentages vs. concentrations of the three tested LEO samples and Trolox, which was used as the positive control (EC_50_ = 1002.0 µM) (Supplementary Materials Figure S3). The Trolox equivalent antioxidant capacity (TEAC) was then estimated and reported in micromoles of Trolox equivalent per milligram of LEO (µmol TE/mg EO). The higher the TEAC value, the stronger the antioxidant activity. Statistical comparisons of the EC_50_ and TEAC means from the technical replicates showed differences between them (*p* values < 0.05). Sample S26 presented the highest antioxidant activity with a TEAC of 23.35 ± 1.32 µmol TE/mg EO (Table 4), followed by samples S36 and S10 (18.76 ± 0.64 and 17.16 ± 0.70 µmol TE/mg EO, respectively). The LEO of *A. rudis* possesses ABTS+• radical scavenging activity.

#### 2.3.3. Anti-Inflammatory Activity

The in vitro anti-inflammatory activity of samples S10, S26 and S36 was evaluated using the lipoxygenase (LOX) inhibition method. LOXs are dioxygenases, key enzymes involved in the biosynthesis of leukotrienes, which are mediators of numerous disorders related to inflammatory processes, such as bronchial asthma, arthritis, and cancer [10]. The discovery of new LOX inhibitors is crucial, as they would prevent the overproduction of leukotrienes and could thus constitute new therapeutic tools for the treatment of human diseases related to inflammation. Inhibition of soybean LOX enzyme was measured and considered a potential indicator of anti-inflammatory activity. Nordihydroguaiaretic acid (NDGA), a non-competitive inhibitor of LOX, was used as the positive control. The three tested LEO samples showed LOX inhibition capacities. The inhibition percentages increased with concentration (Table 5). The concentrations that inhibited 50% of the LOX enzyme (IC_50_) were estimated using linear regression (Supplementary Materials Figure S4). Statistical analyses of the IC_50_ means from the technical replicates revealed differences in the activities of the oil samples between each other and also compared with NDGA (*p* < 0.05). The highest activity was observed for sample S36 (IC_50_: 22.2 ± 1.0 µg/mL), followed by samples S26 and S10, which had similar activities (IC_50_: 32.1 ± 1.0 and 34.9 ± 1.0 µg/mL, respectively). Compared to NDGA (IC_50_: 13.0 ± 1.3 µg/mL), these oils exhibit anti-inflammatory potential.

The antioxidant and anti-inflammatory properties of samples S10, S26 and S36 of *A. rudis* are obviously linked to their chemical composition. Indeed, the tested samples contained, in varying quantities, numerous compounds with various pharmacological and biological properties. These compounds could act synergistically and enhance the activity of the essential oils relative to that of their individual constituents. The main compounds identified in these oils (α-pinene, germacrene D, (*E*)-β-caryophyllene and β-pinene) exhibit antioxidant, anti-inflammatory, antibacterial, antitumor, analgesic and insecticidal activities [12,13,14]. The presence of thymol, a phenolic terpene, in a higher proportion in sample S26 could partly explain its stronger antioxidant activity, as phenolic compounds are recognized antioxidants [15]. Similarly, the more pronounced anti-inflammatory activity of sample S36 could be related to its high contents of germacrene D and (*E*)-β-caryophyllene [16]. The latter is the first dietary cannabinoid approved by the Food and Drug Administration (FDA) and the European Food Safety Authority (EFSA), likely due to its anti-inflammatory activity. This plant compound is also used as a flavor enhancer and in cosmetics [14]. The diverse properties of the major compounds of the LEO of *A. rudis* and their synergistic effects may have contributed to its traditional use in treating parasitic and inflammatory conditions, as well as for healing wounds and burns [8].

### 2.4. Study Limitations

Although this study provides valuable information on the chemical composition and antioxidant and anti-inflammatory activities of the LEO samples obtained from *A. rudis*, no cytotoxicity, stability, microbiological preservation model, or in vivo anti-inflammatory tests have been performed. These evaluations are planned as part of future, more in-depth studies.

## 3. Materials and Methods

### 3.1. Plant Collection and Essential Oil Extraction

The fresh leaves of flowering *A. rudis* were harvested from three locations in southern Côte d’Ivoire: the edge of the Yapo-Abbé Forest, Region of Agneby-Tiassa, geographical coordinates of 5°40′49.6″ N and 4°06′02.6″ W (Location 1; 12 samples); near Agou, Region of Mé, geographical coordinates of 5°58′54.4″ N and 3°56′14.6″ W (Location 2; 12 samples); and Cocody, District of Abidjan, geographical coordinates of 5°20′38.4″ N and 3°59′3.3″ W (Location 3; 12 samples) in Yapo-Abbé Forest (central sampling area is located approximately 39 km North of Cocody and 38 km South of Agou). The harvest took place in November 2024 (rainy season), and January and March 2025 (dry season). The plant material was authenticated by botanists from the Centre National de Floristique (CNF, Abidjan, Côte d’Ivoire) and the Centre Suisse de Recherches Scientifiques (CSRS) in accordance with voucher specimen number LAA5388 available at the CNF. The fresh leaves (500–1200 g) were subjected to hydrodistillation for 3 h using a Clevenger-type apparatus, without a storage step.

### 3.2. Gas Chromatography

Gas chromatography analyses were carried out on a PerkinElmer Clarus 500 Chromatograph (PerkinElmer, Courtaboeuf, France) equipped with two fused-silica capillary columns (50 m × 0.22 mm, film thickness: 0.25 µm), polydimethylsiloxane (BP-1) and polyethylene glycol (BP-20), and a flame ionization detector (FID). The oven temperature program was from 60 °C to 220 °C at a rate of 2 °C/min and then held isothermal at 220 °C for 20 min; injector temperature: 250 °C; detector temperature: 250 °C; carrier gas: (hydrogen, 0.8 mL/min); split ratio: 1/60; injected volume: 0.5 µL. Retention indices (RIs) were calculated relative to the retention times of an *n*-alkanes (C8–C29) series using “Target Compounds” software (appli_kovats2k, 2023) from PerkinElmer (Waltham, MA, USA).

### 3.3. Gas Chromatography–Mass Spectrometry

The LEO samples were analyzed by EI-MS using a PerkinElmer Clarus SQ8S TurboMass detector (quadrupole) directly coupled to a PerkinElmer Clarus 580 Autosystem XL (PerkinElmer, Courtaboeuf, France). The latter was equipped with a polydimethylsiloxane fused-silica capillary column (BP-1: 60 m × 0.22 mm i.d.; film thickness: 0.25 µm). The oven temperature program was from 60 to 230 °C at a rate of 2°/min and then held isothermal for 45 min. Additionally, the injector temperature was 250 °C; the injection volume was 0.2 µL; the ion-source temperature was 250 °C and the ionization energy was 70 eV; the carrier gas was helium (1 mL/min); the split ratio was 1:80. The electron impact (EI) mass spectra were acquired over the mass range of 35–350 Da.

### 3.4. Nuclear Magnetic Resonance

The ^13^C-NMR spectra of the LEO samples were recorded on a Bruker AVANCE 400 spectrometer (Bruker, Wissembourg, France) operating at a frequency of 100.623 MHz for ^13^C and equipped with a 5 mm probe. CDCl_3_ was the solvent used, and all shifts were referred to the internal TMS. The other parameters were as follows: pulse width = 4 µs (flip angle 45°); relaxation delay D1 = 0.1 s; acquisition time = 2.7 s for 128 K data table with a spectral width of 25,000 Hz (250 ppm); CPD decoupling mode; digital resolution = 0.183 Hz/pt. For each LEO sample (40 mg in 0.5 mL of CDCl_3_), 3000 scans were recorded.

### 3.5. Identification of Individual Components

The individual components identification was based on: (i) comparing their GC retention indices (RIs) determined using non-polar and polar capillary columns, relative to the retention times of a series of *n*-alkanes, with those of reference compounds and with reference data [17,18]; (ii) software matching against digital mass spectral libraries [19,20,21]; (iii) comparing the ^13^C-NMR signals with those of reference data compiled in the laboratory-made spectral library using a laboratory-made software [11]. In the investigated LEO samples, compounds at contents as low at 0.4% were identified by NMR.

### 3.6. Antioxidant Activity

The antioxidant capacity of the LEO samples of *A. rudis* was evaluated using DPPH (2,2-diphenyl1-picrylhydrazyl) and ABTS (2,2′-azino-bis(3-ethylbenzothiazoline-6-sulfonic acid)) assays. A Jenway 7315 UV/visible spectrophotometer (Neo-Tech SA, Milmort, Belgium) and a 10 mm quartz cuvette were used for absorbance measurements.

DPPH assay: DPPH• radical solution (100 µM; 0.0395 mg/mL) and various concentrations (31.25, 62.5, 125 and 250 µg/mL) of LEO were prepared in absolute ethanol. Then, 2 mL of the DPPH• solution and 2 mL of each LEO concentration were introduced into separate tubes and, after shaking, were kept protected from light for 30 min. The absorbances of the mixtures were read at 517 nm against a blank consisting of 2 mL of the DPPH• solution and 2 mL of absolute ethanol. Different concentrations of vitamin C (7.81, 15.63, 31.25 and 40.0 µg/mL) were prepared and tested under the same conditions as the essential oils to serve as the positive control. The DPPH• radical inhibition percentages were calculated using Formula (1), and the effective antioxidant concentration required to scavenge 50% of the DPPH• radicals (EC_50_) was determined by plotting the inhibition against sample concentrations in a linear regression [22].

(1) $$\mathrm{Inhibition}\%=\frac{A_{\mathrm{blank}}-A_{\mathrm{sample}}}{A_{\mathrm{blank}}}\times 100$$

A_blank_: absorbance of the blank (in absence of inhibitor); A_sample_: absorbance in presence of inhibitor solution (LEO or positive control solution).

ABTS assay: The ABTS+• radical solution was produced by mixing, in a 1:1 (*v*/*v*) ratio, a 7 mM aqueous solution of ABTS and a 2.45 mM aqueous solution of potassium persulfate (K_2_S_2_O_8_). The mixture was incubated at room temperature (27–30 °C) in the dark for 16 h, after which, the ABTS+• solution was diluted with absolute methanol until it reached an absorbance of 0.70 ± 0.02 at 734 nm. Different concentrations (31.25, 62.5, 125 and 250 mg/L) of LEO were prepared in absolute methanol. Tests were then performed by adding 100 µL of each LEO concentration to 3.9 mL of the diluted ABTS+• solution. After stirring and incubating the mixture for 6 min in the dark, the residual absorbance of the ABTS+• radical was read at 734 nm against a blank consisting of 3.9 mL of the ABTS+• solution and 100 µL of absolute methanol. Calibration was performed by measuring the absorbance of different Trolox concentrations (375.0, 500.0, 625.0, 1000.0, 1125.0, 1375.0, and 1500.0 µM). The inhibition percentages were calculated using formula (1), and the effective antioxidant concentration required to scavenge 50% of the ABTS+• radicals (EC_50_) was determined by plotting the inhibition against the sample concentration using a linear regression. The Trolox Equivalent Antioxidant Capacity (TEAC) was obtained, using Formula (2), as the ratio between the Trolox EC_50_ (µM) and the sample EC_50_ (mg/L) [23,24,25]. The dimensional ratio is (µmol/L)/(mg/L) = µmol/mg; hence, TEAC is expressed as µmol TE per mg of LEO.

(2) $$\mathrm{TEAC}=\frac{{\mathrm{Trolox}\;\mathrm{EC}}_{50}\;}{{\mathrm{Sample}\;\mathrm{EC}}_{50}}$$

### 3.7. In Vitro Anti-Inflammatory Activity

The anti-inflammatory activity of the LEO samples of *A. rudis* was evaluated using the in vitro lipoxygenase (LOX) inhibition assay. Lipoxidase I-B (LOX, EC 1.13.11.12) from soybean was purchased from Sigma-Aldrich (Saint-Quentin-Fallavier, France). It was used to evaluate the LOX inhibitory activity, which was assessed by continuously monitoring the formation of the conjugated dienes of 13-hydroperoxides from linoleic acid at 234 nm, using a spectrophotometric method [26,27,28]. The solution of LOX was prepared by dissolving around 5.7 units/mL of LOX in a Phosphate-Buffer Solution (PBS; 1 unit corresponding to 1 µmol of hydroperoxide formed per min). The LEO samples were diluted in DMSO (dimethyl-sulfoxide) and used as inhibitors of LOX activity. Six concentrations were tested: 12.50, 25.00, 31.25, 50.00, 62.50 and 80.00 µg/mL. The LOX inhibition assays were performed as previously described [28].

Briefly, the LOX solution (10 µL) and the inhibitor solution (10 µL) were mixed in 970 µL of a boric acid buffer (50 mM; pH 9.0). The mixture was incubated briefly at room temperature before starting the reaction by adding 10 µL of linoleic acid at 25 mM (substrate solution). The reaction rate was recorded for 30 s at 234 nm. One measurement was taken in the absence of the inhibitor solution (LEO) and another with DMSO (solvent) mixed with distilled water (99.85% DMSO in distilled water) in order to assess any possible inhibitory effect of DMSO. The LOX activity was not altered by DMSO and the measurement of the LOX activity without the inhibitor solution was used as a negative control (100% enzymatic reaction). All tests were performed with technical replications. The LOX inhibition percentage was calculated using Equation (1). The IC_50_ was determined, using the linear regression equation, as the concentration of the LEO in DMSO that inhibited 50% of the LOX activity.

### 3.8. Statistical Analysis

The chemical profiles of the 36 LEO samples from *A. rudis* were subjected to principal component analysis (PCA) and hierarchical clustering (HC) using XLSTAT 2016 software (Addinsoft, Paris, France) [29]. Constituents at a percentage of 1.0% and higher were used as variables for the HC analysis and PCA. The suitability of the correlation matrix was determined using the Kaiser–Meyer–Olkin criterion. The correlation matrix (Pearson) was used. The data were therefore automatically centered and reduced prior to PCA. The HC and dendrogram were generated using dissimilarity matrices derived from the Euclidean distance. The aggregation method was systematically chosen as the average linkage. One-way analysis of variance (ANOVA) was used to analyze the antioxidant and anti-inflammatory assay results. Tukey (HSD), Bonferroni, and Dunnett (bilateral) tests were used to evaluate the differences between the mean values of EC_50_, TEAC and IC_50_ of the oil samples tested and the positive controls, with a threshold of 5% (*p* values < 0.05 considered significant).

## 4. Conclusions

The LEO of Ivorian *A. rudis* has been investigated for the first time. The detailed analysis of 36 LEO samples, carried out using a combination of chromatographic [GC(RI)] and spectroscopic (GC-MS, ^13^C-NMR) techniques, allowed for the identification of 58 components. The compositions were dominated by mono-and sesquiterpene hydrocarbons (mainly α-pinene, β-pinene, germacrene D and (*E*)-β-caryophyllene), and statistical analyses revealed chemical variability in three clusters. Two main points can be highlighted from these results. First, the identified major components were representative of the diversity of *A. rudis* LEO. Second, the observed chemical differences were associated with locations.

The measurement of antioxidant capacity has revealed that the LEO of *A. rudis* exhibits interesting activities in DPPH and ABTS assays.

The low ratio between the three IC_50_ values (LEO *of A. rudis* vs. NDGA) in the anti-inflammatory test indicates that the LEO of *A. rudis* could be a potent inhibitor of LOX activity [30]. Thus, based on the results, the LEO of *A. rudis* exhibits anti-inflammatory potential.

## Figures and Tables

**Table 1:** Chemical variability of the main constituents of LEO samples from *A. rudis*.

Compound [a]	RIa [b]	RIp [b]	Cluster I			Cluster II			Cluster III		
			M% ± SD	Min	Max	M% ± SD	Min	Max	M% ± SD	Min	Max
α-Pinene	932	1021	24.0 ± 2.9	19.6	28.8	38.9 ± 2.4	36.0	43.1	51.9 ± 5.3	46.6	59.8
β-Pinene	971	1117	5.8 ± 1.3	3.9	8.2	9.3 ± 1.9	6.8	12.8	11.7 ± 1.7	9.3	13.5
Limonene	1021	1205	1.5 ± 0.5	0.9	2.7	2.3 ± 1.5	0.8	5.0	2.9 ± 0.4	2.2	3.6
Thymol	1267	2190	2.4 ± 3.0	0.1	7.5	2.3 ± 2.1	0.1	6.5	2.3 ± 3.3	tr	7.8
(*E*)-β-Caryophyllene	1416	1599	13.1 ± 1.7	11.3	16.8	8.4 ± 1.6	5.9	10.7	3.8 ± 1.3	2.1	5.7
α-Humulene	1449	1670	5.2 ± 1.1	3.6	7.1	3.0 ± 1.0	1.3	5.0	2.0 ± 0.8	1.2	3.5
Germacrene D	1475	1711	27.2 ± 2.7	20.5	30.8	19.1 ± 3.5	13.2	23.6	10.7 ± 2.2	7.3	13.7

[a] Order of elution; [b] RIa, RIp: Retention indices measured on non-polar and polar columns, respectively; M% ± SD: mean percentage and standard deviation; tr: traces (<0.05%).

**Table 2:** Chemical composition (mean) of three LEO samples from *A. rudis*.

N°	Compound	RIa	RIp	S10 (C III)	S26 (C II)	S36 (C I)	Identification Mode
1	α-Thujene	923	1022	1.3	0.5	0.3	RI, MS, ^13^C-NMR
2	α-Pinene	932	1021	51.0	36.0	24.4	RI, MS, ^13^C-NMR
3	Camphene	944	1071	0.2	0.1	0.1	RI, MS
4	Oct-1-en-3-ol	963	1453	tr	0.2	0.2	RI, MS
5	Sabinene	966	1127	1.3	1.0	0.8	RI, MS, ^13^C-NMR
6	β-Pinene	971	1117	10.1	11.7	5.3	RI, MS, ^13^C-NMR
7	Myrcene	981	1166	1.5	1.1	1.3	RI, MS, ^13^C-NMR
8	α-Phellandrene	997	1176	0.3	tr	0.1	RI, MS
9	*p*-Cymene	1012	1277	0.1	tr	0.1	RI, MS
10	β-Phellandrene *	1021	1214	2.4	0.3	1.2	RI, MS, ^13^C-NMR
11	Limonene *	1021	1205	2.9	0.8	1.9	RI, MS, ^13^C-NMR
12	(*Z*)-β-Ocimene	1025	1238	0.1	0.1	0.2	RI, MS
13	(*E*)-β-Ocimene	1036	1255	0.3	1.3	1.2	RI, MS, ^13^C-NMR
14	γ-Terpinene	1048	1250	0.2	0.1	tr	RI, MS
15	Linalool	1086	1550	0.1	tr	tr	RI, MS
16	(*E*)-4,8-Dimethyl, 1,3,7-nonatriene	1105	1311	0.2	0.1	0.1	RI, MS
17	*trans*-Verbenol	1130	1676	0.5	0.1	0.1	RI, MS, ^13^C-NMR
18	Terpinen-4-ol	1162	1599	0.4	0.1	0.1	RI, MS, ^13^C-NMR
19	Thymol	1267	2190	tr	6.5	0.4	RI, MS, ^13^C-NMR
20	δ-Elemene	1335	1472	tr	0.1	0.2	RI, MS
21	α-Ylangene	1370	1484	0.1	0.1	0.1	RI, MS
22	α-Copaene	1374	1493	0.2	0.1	0.1	RI, MS
23	β-Bourbonene	1382	1520	0.1	0.2	0.1	RI, MS
24	β-Elemene *	1386	1592	0.7	1.0	2.1	RI, MS, ^13^C-NMR
25	β-Cubebene *	1386	1540	0.2	0.1	0.1	RI, MS
26	(*E*)-Cinnamyl acetate	1408	2152	-	0.2	tr	RI, MS
27	(*E*)-β-Caryophyllene	1416	1599	5.7	6.5	13.5	RI, MS, ^13^C-NMR
28	Valerena-4,7(11)-diene	1425	1590	0.1	0.1	0.2	RI, MS
29	γ-Elemene #	1426	1640	0.3	0.2	0.8	RI, MS, ^13^C-NMR
30	α-Guaïene	1434	1670	-	tr	tr	RI, MS
31	(*E*)-β-Farnesene	1446	1670	0.1	-	0.2	RI, MS
32	α-Humulene	1449	1670	2.7	2.8	5.1	RI, MS, ^13^C-NMR
33	γ-Muurolene	1469	1689	tr	0.2	0.2	RI, MS
34	Germacrene D	1475	1711	11.5	21.6	29.8	RI, MS, ^13^C-NMR
35	β-Selinene	1480	1719	0.1	0.1	0.1	RI, MS
36	4-*epi*-Cubebol	1486	1886	0.1	0.2	0.1	RI, MS
37	Bicyclogermacrene	1489	1733	0.3	0.5	0.7	RI, MS, ^13^C-NMR
38	α-Muurolene	1491	1724	0.1	0.2	0.2	RI, MS
39	(*E*,*E*)-α-Farnesene	1494	1751	0.1	0.3	0.4	RI, MS
40	β-Bisabolene	1499	1729	0.1	0.3	0.2	RI, MS
41	γ-Cadinene	1504	1758	0.4	1.2	0.2	RI, MS, ^13^C-NMR
42	δ-Cadinene	1513	1758	0.8	0.5	0.6	RI, MS, ^13^C-NMR
43	β-Elemol	1533	2079	tr	0.2	0.2	RI, MS
44	(*E*)-Nerolidol	1546	2042	0.2	0.2	0.3	RI, MS
45	Germacrene B #	1549	1827	0.5	0.4	1.9	RI, MS, ^13^C-NMR
46	Spathulenol	1562	2121	0.1	0.2	0.2	RI, MS
47	Caryophyllene oxide	1569	1979	0.4	0.2	0.6	RI, MS, ^13^C-NMR
48	Humulene oxide II	1591	2035	0.3	0.1	0.2	RI, MS
49	*epi*-Cubenol	1606	2048	tr	0.3	tr	RI, MS
50	Alismol	1609	2253	0.1	-	0.1	RI, MS
51	1,10-*diepi*-Cubenol	1616	2055	-	0.1	0.1	RI, MS
52	τ-Cadinol	1625	2168	-	tr	tr	RI, MS
53	τ-Muurolol	1626	2182	tr	tr	-	RI, MS
54	β-Himachalol	1633	2216	0.6	0.1	0.2	RI, MS, ^13^C-NMR
55	α-Cadinol	1636	2227	0.1	tr	0.3	RI, MS
56	Cadina-1(10),4-dien-8β-ol	1674	2283	-	0.1	0.4	RI, MS
57	Benzyl benzoate	1721	2620	tr	0.1	0.3	RI, MS
58	(*E*)-Phytol	2096	2610	0.2	0.2	1.3	RI, MS, ^13^C-NMR
	Monoterpene hydrocarbons			71.9	53.1	37.0	
	Oxygenated monoterpenes			1.0	6.9	0.6	
	Sesquiterpene hydrocarbons			24.1	36.5	56.8	
	Oxygenated sesquiterpenes			1.9	1.7	2.7	
	Other compounds			0.2	0.5	1.8	
	Total identified			99.1	98.7	98.9	

Elution order and percentages are from a non-polar column (BP-1), except for components marked with an asterisk (*), whose percentages were measured from a polar column (BP-20). (#) Thermolabile component, percentage calculated through a combination of GC(FID) and ^13^C-NMR. RIa, RIp: retention indices measured on non-polar and polar columns, respectively. (-): not detected; tr: traces (<0.05%).

**Table 3:** DPPH radical scavenging capacity of LEO samples from *A. rudis*.

LEO Concentration (µg/mL)	Inhibition (%)		
	S10	S26	S36
31.25	46.152 ± 0.170	48.223 ± 0.078	46.546 ± 0.078
62.50	53.012 ± 0.078	55.670 ± 0.078	54.235 ± 0.135
125.00	66.951 ± 0.135	70.927 ± 0.078	68.004 ± 0.078
250.00	83.470 ± 0.135	86.456 ± 0.135	84.141 ± 0.078
EC_50_ (µg/mL)	43.8 ± 1.0	28.5 ± 1.0	38.8 ± 1.0
	Vitamin C = 15.8 ± 1.0		

Values are means of technical triplicates ± standard deviation.

**Table 4:** ABTS cation radical scavenging by LEO samples from *A. rudis*.

LEO Concentration (mg/L)	Inhibition (%)		
	S10	S26	S36
31.25	44.324 ± 0.098	46.014 ± 0.135	45.208 ± 0.078
62.50	52.127 ± 0.078	53.419 ± 0.078	52.110 ± 0.098
125.00	60.020 ± 0.078	63.520 ± 0.098	61.236 ± 0.135
250.00	74.113 ± 0.135	75.132 ± 0.078	74.464 ± 0.098
EC_50_ (mg/L)	58.4 ± 1.0	42.9 ± 1.0	53.4 ± 1.0
TEAC (µmol TE/mg EO)	17.16 ± 0.70	23.35 ± 1.32	18.76 ± 0.64

Values are means of technical triplicates ± standard deviation.

**Table 5:** In vitro anti-inflammatory activity of LEO samples from *A. rudis*.

LEO Concentration (µg/mL)	(LOX Inhibition Percentage)		
	S10	S26	S36
12.50	27.41 ± 1.54	32.20 ± 1.28	40.01 ± 1.84
25.00	38.94 ± 1.01	41.61 ± 1.00	52.48 ± 1.59
31.25	47.56 ± 0.52	52.25 ± 1.36	60.52 ± 1.02
50.00	66.40 ± 0.95	65.34 ± 1.64	72.13 ± 0.72
62.50	79.03 ± 0.89	77.71 ± 1.37	85.60 ± 0.67
80.00	90.09 ± 0.53	92.21 ± 0.56	98.33 ± 1.09
IC_50_ (µg/mL)	34.9 ± 1.0	32.1 ± 1.0	22.2 ± 1.0
	NDGA	13.0 ± 1.3	

Values are means of technical triplicates ± standard deviation.

## Data Availability

All the data produced and used in this article are available in the manuscript or in the Supplementary Materials.

## Supplementary Materials

The following supporting information can be downloaded at: https://www.mdpi.com/article/10.3390/plants15060865/s1. Table S1: Plant material and essential oil extraction data; Table S2: Chemical composition of the 36 LEO samples from *Aspilia rudis*; Table S3: Kaiser–Meyer–Olkin (KMO) criterion values; Table S4: Principal component eigenvalues and variances (PCA); Figure S1: Scree plot of the principal components (PCA); Figure S2: Linear regressions of the DDPH antioxidant assay; Figure S3: Linear regressions of the ABTS antioxidant assay; Figure S4: Linear regressions of the LOX inhibition assay.
