# Supplementary material for: Chemical Profile and Related Antioxidant and Anti-Inflammatory Activities of Leaf Essential Oil from Aspilia rudis Oliv. & Hiern
Source: Plants (Basel). 2026 Mar 11;15(6):865. doi: 10.3390/plants15060865 (PMC13029662; doi:10.3390/plants15060865)
Supplement: Supplementary file 1 [file plants-15-00865-s001.zip › plants-4159853-supplementary.pdf]

Supplementary Materials:

Chemical Profile and Related Antioxidant and Anti-inflammatory Activities of Leaf Essential Oil from *Aspilia rudis* Oliv. & Hiern.

Didjour Albert Kambiré [1], Guy Blanchard Boué [2], Mathieu Paoli [3], Ange Bighelli [3], Jean Brice Boti [4], Zanahi Félix Tonzibo [4] and Félix Tomi [3,*]

[1] UPR de Chimie, Département de Mathématiques, Physique et Chimie, UFR des Sciences Biologiques, Université Peleforo Gon Coulibaly, BP 1328 Korhogo, Côte d'Ivoire; dakambire@gmail.com
[2] UFR des Sciences et Technologies, Université Alassane Ouattara, BP V18 Bouaké 01, Côte d'Ivoire; blanchardboue@uao.edu.ci
[3] Laboratoire Sciences Pour l'Environnement, Université de Corse—CNRS, UMR 6134 SPE, Route des Sanguinaires, 20000 Ajaccio, France; paoli_m@univ-corse.fr (M.P.); bighelli_a@univ-corse.fr (A.B.)
[4] Laboratoire de Constitution et Réaction de la Matière, UFR-SSMT, Université Félix Houphouët-Boigny, BP V34 Abidjan, Côte d'Ivoire; jeanbriceboti@hotmail.fr (J.B.B.); tonzibz@yahoo.fr (Z.F.T.)
* Correspondence: tomi_f@univ-corse.fr

Abstract

The present study reports for the first time the chemical composition of the leaf essential oil (LEO) from *Aspilia rudis* as well as its antioxidant and anti-inflammatory activities. Analysis by combination of GC(RI), GC-MS and ^{13}C-NMR of 36 samples identified 58 compounds representing 96.7–99.3% of the whole composition. Statistical analyses revealed a chemical variability though 3 clusters, each composed of samples from the same sampling site. Cluster I is dominated by germacrene D (27.2 ± 2.7%), α-pinene (24.0 ± 2.9%) and (*E*)-β-caryophyllene (13.1 ± 1.7%), cluster II by α-pinene (38.9 ± 2.4%) and germacrene D (19.1 ± 3.6%), while the prevalent compound of cluster III is α-pinene (51.9 ± 5.3%), followed by β-pinene (11.7 ± 1.7%) and germacrene D (10.7 ± 2.2%). The oil samples S10, S26 and S36 demonstrated antioxidant activity (DPPH: EC_{50} = 43.8 ± 1.0, 28.5 ± 1.0 and 38.8 ± 1.0 µg/mL, respectively; ABTS: TEAC = 17.16 ± 0.70, 23.35 ± 1.32 and 18.76 ± 0.64 µmol TE/mg EO, respectively) and anti-inflammatory activity through the LOX inhibition assay (IC_{50} = 34.9 ± 1.0, 32.1 ± 1.0 and 22.2 ± 1.0 µg/mL, respectively). The activities of *A. rudis* LEO may be related to its main compounds and thymol, all known for their various pharmacological and biological properties, and probably to potential synergistic effects.

Keywords: *Aspilia rudis*, leaf oil, chemical composition, antioxidant, anti-inflammatory

Table S1: Plant material and essential oil extraction data.

Table S2: Chemical composition of the 36 leaf essential oil samples from *Aspilia rudis*.

Table S3: Kaiser-Meyer-Olkin (KMO) criterion values.

Table S4: Principal components eigenvalues and variances (PCA).

Figure S1: Scree plot of the principal components (PCA).

Figure S2: Linear regressions of the DDPH antioxidant assay.

Figure S3: Linear regressions of the ABTS antioxidant assay.

Figure S4: Linear regressions of the LOX inhibition assay.

Table S1. Plant material and essential oil extraction data.

Samples	Leaves weight (g)	Essential oil weight (mg)	Extraction yield (%)	Harvest site	Month	Season
S1	983.4	1288.3	0.131	Location 1	November	Rainy
S2	922.2	1512.4	0.164	Location 1	November	Rainy
S3	753.1	1378.1	0.183	Location 1	November	Rainy
S4	1112.0	2457.6	0.221	Location 1	November	Rainy
S5	1012.3	1508.3	0.149	Location 2	November	Rainy
S6	1012.8	1620.5	0.160	Location 2	November	Rainy
S7	945.9	1579.7	0.167	Location 2	November	Rainy
S8	1193.2	1587	0.133	Location 2	November	Rainy
S9	965.4	1911.4	0.198	Location 3	November	Rainy
S10	1250.4	2800.8	0.224	Location 3	November	Rainy
S11	1061.3	1422.2	0.134	Location 3	November	Rainy
S12	1125.0	1980	0.176	Location 3	November	Rainy
S13	806.0	1023.6	0.127	Location 3	January	Dry
S14	1173.4	1936.1	0.165	Location 3	January	Dry
S15	856.4	1806.9	0.211	Location 3	January	Dry
S16	651.6	1257.6	0.193	Location 3	January	Dry
S17	790.2	1454	0.184	Location 1	January	Dry
S18	916.5	1613.1	0.176	Location 1	January	Dry
S19	1066.0	1343.2	0.126	Location 1	January	Dry
S20	1089.8	2081.6	0.191	Location 1	January	Dry
S21	945.1	1313.7	0.139	Location 2	January	Dry
S22	893.3	1545.4	0.173	Location 2	January	Dry
S23	693.7	1151.5	0.166	Location 2	January	Dry
S24	1011.6	2185.1	0.216	Location 2	January	Dry
S25	789.3	1554.9	0.197	Location 2	March	Dry
S26	1097.5	2458.5	0.224	Location 2	March	Dry
S27	727.8	1273.7	0.175	Location 2	March	Dry
S28	869.9	1444.1	0.166	Location 2	March	Dry
S29	858.3	1356.1	0.158	Location 3	March	Dry
S30	1152.1	2315.8	0.201	Location 3	March	Dry
S31	654.3	1275.9	0.195	Location 3	March	Dry
S32	688.1	1438.1	0.209	Location 3	March	Dry
S33	881.9	1666.7	0.189	Location 1	March	Dry
S34	923.8	1672	0.181	Location 1	March	Dry
S35	576.8	1257.5	0.218	Location 1	March	Dry
S36	1197.7	2670.8	0.223	Location 1	March	Dry

Location 1: Edge of the Yapo-Abbé Forest, Region of Agneby-Tiassa (5°40'49.6"N and 4°06'02.6"W); Location 2: Agou, Region of Mé (5°58′54.4″ N and 3°56′14.6″ W) and Location 3: Cocody, District of Abidjan (5°20′38.4″ N and 3°59′3.3″ W).

Table S2. Chemical composition of the 36 leaf essential oil samples from *Aspilia rudis* (Clusters I and II)

N°	Compounds	RIa	RIp	Cluster I												Cluster II												Identification mode
				S1	S2	S3	S4	S17	S18	S19	S20	S33	S34	S35	S36	S5	S6	S7	S8	S21	S22	S23	S24	S25	S26	S27	S28	
1	α-Thujene	923	1 022	0.1	0.2	0.3	0.4	0.2	0.2	0.1	0.1	0.2	0.1	0.1	0.3	0.2	0.1	0.1	0.8	0.7	tr	tr	0.1	tr	0.5	1.0	0.1	RI, MS, ^{13}C-NMR
2	α-Pinene	932	1 021	26.7	28.8	26.0	25.9	20.1	23.0	20.5	19.6	23.0	24.9	25.5	24.4	37.0	37.8	37.5	36.3	38.7	38.5	39.1	41.8	43.1	36.0	42.8	38.3	RI, MS, ^{13}C-NMR
3	Camphene	944	1 071	0.1	0.1	0.2	0.1	0.1	0.1	0.1	0.1	0.2	0.2	0.1	0.1	0.2	0.2	0.2	0.2	0.1	0.1	0.1	0.2	0.1	0.1	0.1	0.1	RI, MS
4	Oct-1-en-3-ol	963	1 453	0.4	0.3	0.2	tr	-	-	0.1	0.1	0.2	0.3	0.1	0.2	0.3	0.1	0.1	0.2	-	0.1	0.1	tr	0.1	0.2	0.5	-	RI, MS, ^{13}C-NMR
5	Sabinene	966	1 127	0.8	1.3	0.9	0.5	0.5	0.6	0.6	0.4	0.9	1.0	1.2	0.8	0.9	0.9	1.0	1.2	0.8	0.6	0.6	1.5	1.1	2.7	2.5	1.5	RI, MS, ^{13}C-NMR
6	β-Pinene	971	1 117	5.3	6.5	8.2	5.1	3.9	5.3	5.5	4.1	7.6	7.1	6.2	5.3	6.8	7.3	7.6	8.2	7.6	9.3	8.7	12.8	10.1	11.7	11.5	9.9	RI, MS, ^{13}C-NMR
7	Myrcene	981	1 166	1.5	1.5	1.5	0.6	0.9	1.1	1.6	0.6	1.7	1.4	1.7	1.3	1.9	1.8	1.9	1.7	1.0	1.8	1.6	1.9	1.7	1.6	1.7	2.1	RI, MS, ^{13}C-NMR
8	α-Phellandrene	997	1176	0.1	tr	0.1	tr	0.4	0.6	0.1	0.1	0.1	0.1	0.1	0.1	0.1	0.1	0.1	0.1	0.2	tr	0.3	0.4	0.1	tr	0.1	tr	RI, MS, ^{13}C-NMR
9	*p*-Cymene	1 012	1 277	0.1	0.1	tr	0.1	0.1	0.1	0.3	0.2	0.2	0.1	tr	0.1	tr	0.1	-	0.2	0.5	0.1	0.1	0.1	0.1	tr	-	0.1	RI, MS, ^{13}C-NMR
10	β-Phellandrene*	1 021	1 214	1.7	0.3	1.5	0.8	0.6	0.5	0.9	0.4	1.5	1.3	1.4	1.2	1.9	1.9	1.8	2.2	0.6	0.3	1.1	3.7	0.1	0.3	0.3	0.8	RI, MS, ^{13}C-NMR
11	Limonene*	1 021	1 205	1.5	1.0	1.8	0.9	1.7	1.2	1.4	1.5	2.7	1.0	0.9	1.9	1.7	1.5	1.6	1.2	2.6	3.1	4.8	5.0	3.6	0.8	0.8	1.0	RI, MS, ^{13}C-NMR
12	(*Z*)-β-Ocimene	1 025	1 238	0.1	0.1	0.1	0.1	0.1	0.1	0.1	tr	0.2	0.1	0.1	0.2	0.2	0.2	0.1	0.1	tr	0.1	0.1	tr	tr	0.1	0.1	0.2	RI, MS
13	(*E*)-β-Ocimene	1 036	1 255	0.8	1.2	0.6	0.3	0.5	0.5	0.6	0.4	1.1	0.9	1.4	1.2	1.0	1.0	0.9	0.5	1.4	1.5	0.4	0.3	0.4	1.6	1.5	1.4	RI, MS, ^{13}C-NMR
14	γ-Terpinene	1 048	1 250	tr	tr	0.1	0.1	0.2	0.1	0.1	0.1	0.1	0.1	0.1	tr	0.1	tr	0.1	0.1	0.2	0.1	0.1	0.1	0.1	0.1	0.1	0.1	RI, MS
15	Linalool	1 086	1 550	0.1	-	-	0.1	-	tr	0.1	0.1	tr	tr	0.1	tr	-	tr	0.1	-	0.1	0.1	0.2	0.1	0.1	tr	0.1	0.1	RI, MS
16	(*E*)-4,8-Dimethyl, 1,3,7-nonatriene	1 105	1 311	0.1	0.1	0.1	0.1	0.1	0.1	0.1	0.1	0.1	0.1	0.1	0.1	0.1	0.1	0.1	0.1	0.2	0.1	0.1	0.1	0.3	0.1	0.1	0.1	RI, MS
17	*trans*-Verbenol	1 130	1 676	0.1	tr	0.1	0.7	0.2	0.2	0.2	0.1	0.1	0.1	0.1	0.1	0.1	0.1	0.1	0.1	0.2	tr	0.1	0.1	0.2	0.1	0.1	tr	RI, MS, ^{13}C-NMR
18	Terpinen-4-ol	1 162	1 599	0.1	0.1	0.1	0.2	0.3	0.1	0.2	0.2	0.1	0.1	0.1	0.1	0.1	0.1	0.1	0.1	0.9	0.1	1.3	0.4	0.3	0.1	0.2	0.1	RI, MS, ^{13}C-NMR
19	Thymol	1 267	2 190	0.4	0.1	0.5	0.5	6.8	3.3	7.5	7.2	1.3	0.6	0.4	0.4	0.3	0.2	0.1	0.9	2.4	3.3	5.5	2.5	1.8	1.7	0.1	3.5	RI, MS, ^{13}C-NMR
20	δ-Elemene	1 335	1 472	0.2	0.2	0.3	0.1	0.2	0.1	0.1	0.2	0.2	0.2	0.1	0.2	0.2	0.1	0.2	0.2	0.1	0.1	0.2	-	0.1	0.1	0.1	0.1	RI, MS
21	α-Ylangene	1 370	1 484	0.1	0.2	0.1	0.1	0.1	0.1	0.1	0.2	tr	tr	0.1	0.1	0.1	tr	tr	0.1	0.1	0.2	tr	-	0.1	0.1	0.2	0.1	RI, MS
22	α-Copaene	1 374	1 493	0.2	0.4	0.1	0.2	0.2	0.2	0.1	0.3	0.1	0.2	0.2	0.1	0.1	0.1	0.1	0.1	0.4	0.1	0.2	0.1	0.1	0.1	0.2	0.1	RI, MS
23	β-Bourbonene	1 382	1 520	0.1	0.3	0.3	0.1	0.1	0.8	0.1	0.9	0.2	0.1	0.2	0.1	0.1	0.1	0.1	0.2	0.1	0.2	0.1	tr	0.2	0.2	0.2	0.2	RI, MS, ^{13}C-NMR
24	β-Elemene*	1 386	1 592	2.1	1.2	1.6	1.9	0.9	1.7	1.4	1.8	2.6	2.5	2.9	2.1	1.9	1.8	1.8	1.2	1.5	0.7	1.0	0.8	1.0	1.0	0.9	0.8	RI, MS, ^{13}C-NMR
25	β-Cubebene*	1 386	1 540	0.2	0.3	0.1	0.2	0.2	0.2	0.1	0.2	0.1	0.2	0.2	0.1	0.2	0.2	0.2	0.1	0.2	0.2	0.2	0.1	0.2	0.1	0.2	0.1	RI, MS
26	(*E*)-Cinnamyl acetate	1 408	2 152	-	0.1	-	tr	0.1	0.2	0.2	0.3	tr	-	0.1	tr	-	-	-	-	0.1	0.9	-	-	0.1	0.2	0.1	0.6	RI, MS, ^{13}C-NMR
27	(*E*)-β-Caryophyllene	1 416	1 599	13.1	12.0	11.4	12.5	11.3	15.9	12.8	16.8	12.8	12.4	12.5	13.5	10.7	10.0	9.2	9.8	8.1	10.4	7.2	8.8	5.9	6.5	7.7	6.6	RI, MS, ^{13}C-NMR
28	Valerena-4,7(11)-diene	1 425	1 590	tr	1.0	0.3	tr	tr	0.9	tr	tr	0.4	0.2	tr	0.2	tr	0.2	tr	tr	tr	0.2	tr	tr	tr	0.1	0.5	0.1	RI, MS

29	γ-Elemene#	1 426	1 640	0.6	0.3	0.7	0.9	1.1	0.4	0.4	1.2	0.8	0.9	0.4	0.8	0.6	0.5	0.6	0.5	0.5	0.1	0.5	tr	0.5	0.2	tr	0.2	RI, MS, ^{13}C-NMR
30	α-Guaïene	1 434	1 670	tr	0.2	0.1	0.2	0.2	0.2	tr	0.2	tr	tr	0.1	tr	tr	tr	tr	tr	0.5	0.1	0.1	0.1	0.1	tr	0.1	tr	RI, MS, ^{13}C-NMR
31	(*E*)-β-Farnesene	1 446	1 670	0.2	0.1	0.2	0.2	0.2	0.1	0.1	0.1	0.1	0.1	0.1	0.2	0.2	0.2	0.2	0.2	0.1	0.1	0.1	0.5	0.1	-	-	0.1	RI, MS, ^{13}C-NMR
32	α-Humulene	1 449	1 670	4.4	3.9	3.6	7.1	6.6	5.3	4.2	5.9	4.9	5.0	5.9	5.1	3.4	3.5	3.2	3.0	5.0	3.8	3.1	1.3	1.6	2.8	2.9	1.8	RI, MS, ^{13}C-NMR
33	γ-Muurolene	1 469	1 689	0.2	0.3	0.2	0.4	0.3	0.3	0.3	0.3	0.2	0.2	0.2	0.2	tr	0.2	0.2	0.2	0.1	0.2	0.1	0.1	0.1	0.2	0.2	0.2	RI, MS
34	Germacrene D	1 475	1 711	27.8	27.2	27.9	26.3	26.5	25.6	25.0	20.5	28.9	30.8	29.6	29.8	23.2	21.7	23.6	21.0	17.2	14.0	15.2	13.2	18.9	22.6	18.7	20.4	RI, MS, ^{13}C-NMR
35	β-Selinene	1 480	1 719	0.1	0.1	0.2	0.2	0.2	0.2	0.2	0.2	0.2	0.2	0.1	0.1	0.1	0.1	0.1	0.4	0.3	0.1	tr	0.6	0.1	0.1	0.1	0.1	RI, MS, ^{13}C-NMR
36	4-*epi*-Cubebol	1 486	1 886	0.1	0.4	0.1	0.1	0.1	0.1	0.1	0.2	0.1	0.2	0.3	0.1	0.1	0.1	0.1	0.1	0.1	0.1	0.2	-	0.1	0.2	0.1	0.1	RI, MS
37	Bicyclogermacrene	1 489	1 733	0.7	0.6	0.7	0.7	0.8	0.7	0.7	0.7	0.2	0.1	0.5	0.7	0.6	0.6	0.6	0.6	0.2	0.4	0.1	0.2	0.5	0.5	0.5	0.5	RI, MS, ^{13}C-NMR
38	α-Muurolene	1 491	1 724	0.2	0.3	0.2	0.3	0.3	0.3	0.2	0.3	0.1	0.2	0.2	0.2	0.2	0.2	0.2	0.2	0.1	0.1	0.2	tr	0.1	0.2	0.2	0.1	RI, MS
39	(*E,E*)-α-Farnesene	1 494	1 751	0.4	0.9	0.3	0.3	0.5	0.3	0.3	0.5	0.2	0.1	0.4	0.4	0.3	0.2	0.2	0.2	0.1	0.4	0.1	0.1	0.2	0.3	0.1	0.4	RI, MS, ^{13}C-NMR
40	β-Bisabolene	1 499	1 729	0.3	0.4	0.3	0.2	0.3	0.2	0.2	0.3	0.1	0.2	0.3	0.2	0.2	0.2	0.2	0.3	0.2	0.3	0.2	tr	0.2	0.3	0.1	0.2	RI, MS, ^{13}C-NMR
41	γ-Cadinene	1 504	1 758	0.6	2.1	0.1	0.1	0.7	0.6	0.6	1.1	0.1	0.2	0.1	0.2	0.1	0.4	0.5	0.4	tr	1.1	0.8	tr	0.4	1.2	0.9	1.0	RI, MS, ^{13}C-NMR
42	δ-Cadinene	1 513	1 758	0.9	1.1	0.5	1.0	0.8	0.7	0.7	1.0	1.0	1.0	0.6	0.6	0.8	0.8	0.8	0.6	1.4	1.2	1.8	0.6	1.9	0.5	0.5	0.5	RI, MS, ^{13}C-NMR
43	β-Elemol	1 533	2 079	0.2	0.2	0.5	0.1	0.3	0.3	0.6	0.6	0.2	0.1	0.1	0.2	0.1	0.1	0.1	0.3	tr	0.3	0.1	tr	0.2	0.2	tr	0.1	RI, MS, ^{13}C-NMR
44	(*E*)-Nerolidol	1 546	2 042	0.4	0.2	0.4	0.3	tr	0.2	0.4	0.3	0.1	0.1	0.2	0.3	0.2	0.3	0.2	0.3	0.1	0.1	0.2	0.1	0.1	0.2	0.1	0.2	RI, MS
45	Germacrene B#	1 549	1 827	1.5	0.7	1.6	1.9	2.2	0.9	1.6	2.0	1.9	1.6	1.7	1.9	1.1	1.1	1.1	0.8	1.0	0.4	0.2	tr	0.9	0.4	0.2	0.6	RI, MS, ^{13}C-NMR
46	Spathulenol	1 562	2 121	0.2	0.1	0.2	tr	tr	0.2	0.4	0.3	0.1	0.1	0.2	0.2	tr	0.2	0.2	0.1	0.1	0.3	0.1	0.1	tr	0.2	0.1	0.2	RI, MS
47	Caryophyllene oxide	1 569	1 979	0.6	0.3	0.4	2.0	0.8	0.9	1.2	1.0	0.2	0.3	tr	0.6	0.4	0.5	0.4	0.4	1.4	0.5	0.4	0.7	0.3	0.2	0.2	0.4	RI, MS, ^{13}C-NMR
48	Humulene oxide II	1 591	2 035	0.2	0.1	0.2	0.9	0.4	0.3	tr	0.4	0.1	0.1	0.1	0.2	0.1	0.1	0.1	0.2	0.1	tr	0.2	0.1	0.1	0.1	tr	0.1	RI, MS, ^{13}C-NMR
49	*epi*-Cubenol	1 606	2 048	0.2	0.2	0.3	0.1	0.2	0.2	0.4	0.3	0.1	0.2	0.1	tr	0.1	tr	0.1	0.4	0.1	tr	0.1	tr	tr	0.3	0.1	0.2	RI, MS
50	Alismol	1 609	2 253	0.2	0.1	0.2	0.2	0.3	0.1	0.2	0.2	0.1	0.2	0.1	0.1	0.1	0.1	0.1	0.1	0.1	0.1	0.2	0.1	0.1	-	-	0.1	RI, MS
51	1,10-*diepi*-Cubenol	1 616	2 055	0.1	0.1	0.1	0.1	0.1	0.1	0.1	0.1	0.1	0.1	0.1	0.1	tr	0.1	tr	0.1	0.1	tr	0.1	tr	tr	0.1	tr	0.1	RI, MS
52	τ-Cadinol	1 625	2 168	0.1	0.1	tr	0.2	0.4	tr	0.4	tr	-	0.1	0.1	tr	0.1	0.1	tr	0.1	0.5	tr	0.2	tr	0.1	tr	-	0.1	RI, MS, ^{13}C-NMR
53	τ-Muurolol	1 626	2 182	0.1	0.1	0.2	0.1	0.1	0.2	0.1	0.1	0.2	0.1	-	-	0.1	0.2	0.2	0.3	tr	0.1	0.1	-	tr	tr	tr	0.1	RI, MS
54	β-Himachalol	1 633	2 216	0.1	0.1	0.5	-	0.1	0.1	0.1	0.1	0.2	0.2	0.1	0.2	tr	0.1	tr	0.3	0.1	tr	0.1	-	tr	0.1	0.1	tr	RI, MS, ^{13}C-NMR
55	α-Cadinol	1 636	2 227	0.4	0.2	0.4	tr	0.5	0.2	0.4	0.4	0.1	0.1	tr	0.3	0.2	0.3	0.3	0.2	0.1	tr	0.2	-	0.1	tr	tr	tr	RI, MS, ^{13}C-NMR
56	Cadina-1(10),4-dien-8β-ol	1 674	2 283	0.1	tr	0.2	0.8	0.4	0.2	0.2	0.4	-	-	0.1	0.4	tr	0.2	0.1	0.1	0.1	0.1	0.1	0.1	0.1	0.1	-	0.1	RI, MS, ^{13}C-NMR
57	Benzyl benzoate	1 721	2 620	tr	tr	0.1	0.2	2.4	1.2	2.9	2.7	tr	tr	0.1	0.3	tr	tr	tr	0.1	0.2	2.4	tr	tr	0.7	1.0	0.1	1.9	RI, MS, ^{13}C-NMR
58	(*E*)-Phytol	2 096	2 610	2.1	1.4	1.3	0.6	1.2	0.3	1.3	0.7	1.3	1.1	1.4	1.3	0.8	0.7	0.6	1.2	0.4	0.7	1.3	0.4	0.2	0.6	0.1	0.9	RI, MS, ^{13}C-NMR
	Monoterpene hydrocarbons			38.9	41.2	41.4	35.0	29.4	33.5	32.0	27.7	39.6	38.4	38.9	37.0	52.1	53.0	53.0	52.9	54.6	55.6	57.1	68.0	60.8	55.6	62.6	55.7	
	Oxygenated monoterpenes			0.7	0.3	0.7	1.5	7.4	3.8	8.2	7.9	1.5	0.8	0.8	0.6	0.5	0.4	0.4	1.1	3.7	4.4	7.1	3.1	2.5	2.1	0.6	4.3	

Sesquiterpene hydrocarbons	53.9	53.8	50.8	54.9	53.7	55.7	49.2	54.7	55.1	56.4	56.4	56.8	44.1	42.2	43.1	40.1	37.2	34.4	31.4	26.5	33.2	37.5	34.5	34.2
Oxygenated sesquiterpènes	3.0	2.2	3.7	4.9	3.7	3.1	4.6	4.4	1.6	1.9	1.5	2.7	1.5	2.4	1.9	3.0	2.9	1.6	2.3	1.2	1.2	1.7	0.7	1.8
Other compounds	2.5	1.7	1.6	0.8	3.6	1.5	4.3	3.5	1.5	1.4	1.6	1.8	1.1	0.8	0.7	1.5	0.6	3.2	1.4	0.4	1.0	1.8	0.7	2.8
**Total identified**	**99.0**	**99.2**	**98.2**	**97.1**	**97.8**	**97.6**	**98.3**	**98.2**	**99.3**	**98.9**	**99.2**	**98.9**	**99.3**	**98.8**	**99.1**	**98.6**	**99.0**	**99.2**	**99.3**	**99.2**	**98.7**	**98.7**	**99.1**	**98.8**

[a]Order of elution and percentages are given on a non-polar column (BP-1), except components with an asterisk (*), where percentages are taken on a polar column (BP-20). (#) Thermolabile compound, percentage evaluated by a combination of GC-FID and ^{13}C-NMR data. RIa, RIp: retention indices measured on non-polar and polar capillary column, respectively. (-): not detected; tr: traces level (<0.05%).

Table S2 (Continuation). Chemical composition of the 36 leaf essential oil samples from *Aspilia rudis* (Cluster III).

N°	Compounds	RIa	RIp	Cluster III												Identification mode
				S9	S10	S11	S12	S13	S14	S15	S16	S29	S30	S31	S32	
1	α-Thujene	923	1 022	1.1	1.3	0.9	1.3	0.1	0.1	0.1	0.2	0.4	0.6	0.9	0.6	RI, SM, ^{13}C-NMR
2	α-Pinene	932	1 021	48.3	51.0	50.8	49.4	47.1	46.6	46.8	47.4	58.1	59.3	57.9	59.8	RI, SM, ^{13}C-NMR
3	Camphene	944	1 071	0.2	0.2	0.2	0.2	0.1	0.1	0.1	0.2	0.1	0.1	0.1	0.2	RI, SM
4	Oct-1-en-3-ol	963	1 453	tr	tr	-	0.1	0.1	tr	0.2	0.1	0.2	0.2	0.3	-	RI, SM, ^{13}C-NMR
5	Sabinene	966	1 127	1.4	1.3	1.1	1.1	0.7	1.1	0.8	1.1	1.5	1.2	1.6	1.6	RI, SM, ^{13}C-NMR
6	β-Pinene	971	1 117	9.3	10.1	9.4	9.5	13.2	12.2	12.2	12.9	13.5	13.1	13.1	12.1	RI, SM, ^{13}C-NMR
7	Myrcene	981	1 166	1.6	1.5	1.3	1.7	1.7	1.5	1.6	1.6	1.5	1.4	1.4	1.3	RI, SM, ^{13}C-NMR
8	α-Phellandrene	997	1176	0.1	0.3	0.3	0.1	0.5	0.1	tr	0.1	tr	0.1	0.1	0.2	RI, SM, ^{13}C-NMR
9	*p*-Cymene	1 012	1 277	0.1	0.1	0.1	tr	0.1	0.1	-	tr	-	tr	-	0.1	RI, SM, ^{13}C-NMR
10	β-Phellandrene*	1 021	1 214	2.5	2.4	2.3	1.6	0.7	0.6	0.2	0.4	0.3	0.3	0.3	0.5	RI, SM, ^{13}C-NMR
11	Limonene*	1 021	1 205	2.2	2.9	2.6	2.2	3.5	3.6	3.1	3.3	2.8	2.9	2.7	3.1	RI, SM, ^{13}C-NMR
12	(*Z*)-β-Ocimene	1 025	1 238	0.2	0.1	0.1	0.2	0.1	0.1	0.1	0.1	0.1	0.1	0.1	0.1	RI, SM
13	(*E*)-β-Ocimene	1 036	1 255	0.7	0.3	0.4	0.8	0.7	0.8	0.8	0.9	0.5	0.4	0.5	0.3	RI, SM, ^{13}C-NMR
14	γ-Terpinene	1 048	1 250	0.2	0.2	0.1	0.1	0.1	0.2	0.1	0.1	0.1	0.1	0.1	0.3	RI, SM
15	Linalol	1 086	1 550	tr	0.1	tr	-	0.1	0.1	tr	0.1	tr	tr	tr	0.1	RI, SM
16	(*E*)-4,8-Dimethyl, 1,3,7-nonatriene	1 105	1 311	0.1	0.2	0.2	0.1	0.2	0.2	0.1	0.1	0.1	0.1	0.1	0.2	RI, SM
17	*trans*-Verbenol	1 130	1 676	0.2	0.5	0.7	0.1	0.1	0.1	tr	0.1	tr	tr	0.1	0.8	RI, SM, ^{13}C-NMR
18	Terpinen-4-ol	1 162	1 599	0.4	0.4	0.3	0.1	1.1	0.6	0.5	0.8	0.3	0.5	0.4	0.6	RI, SM, ^{13}C-NMR
19	Thymol	1 267	2 190	tr	tr	tr	0.1	5.5	7.8	7.3	6.4	0.1	0.2	0.1	0.1	RI, SM, ^{13}C-NMR
20	δ-Elemene	1 335	1 472	0.1	tr	0.1	0.2	0.1	0.1	0.2	0.3	0.2	0.2	0.2	tr	RI, SM
21	α-Ylangene	1 370	1 484	tr	0.1	tr	0.1	tr	-	0.1	tr	0.1	0.2	0.2	-	RI, SM

22	α-Copaene	1 374	1 493	0.1	0.2	0.1	0.1	0.1	0.2	0.3	0.2	0.1	0.1	0.1	0.1	RI, SM
23	β-Bourbonene	1 382	1 520	0.1	0.1	tr	0.2	0.1	0.1	0.1	0.1	0.2	0.2	0.3	tr	RI, SM, ^{13}C-NMR
24	β-Elemene*	1 386	1 592	0.9	0.7	0.9	1.3	0.5	0.9	0.5	0.4	0.3	0.1	0.3	0.5	RI, SM, ^{13}C-NMR
25	β-Cubebene*	1 386	1 540	0.1	0.2	0.1	0.1	0.1	0.1	0.2	0.1	0.2	0.2	0.2	0.1	RI, SM
26	Cinnamyl acetate	1 408	2 152	-	-	-	-	-	0.2	0.1	-	0.1	0.1	-	-	RI, SM, ^{13}C-NMR
27	(*E*)-β-Caryophyllene	1 416	1 599	4.7	5.7	5.1	5.6	4.3	3.2	3.8	4.2	2.5	2.1	2.1	2.5	RI, SM, ^{13}C-NMR
28	Valerena-4,7(11)-diene	1 425	1 590	tr	0.1	0.1	tr	tr	tr	0.1	tr	0.1	0.1	0.1	tr	RI, SM
29	γ-Elemene#	1 426	1 640	0.5	0.3	0.4	0.7	0.2	0.1	0.3	0.2	0.1	tr	tr	0.2	RI, SM, ^{13}C-NMR
30	α-Guaïene	1 434	1 670	tr	-	tr	0.1	0.1	-	0.2	0.1	0.2	0.1	0.1	-	RI, SM, ^{13}C-NMR
31	(*E*)-β-Farnesene	1 446	1 670	0.1	0.1	0.1	0.1	0.1	0.1	0.1	0.1	tr	0.1	0.1	tr	RI, SM, ^{13}C-NMR
32	α-Humulene	1 449	1 670	3.5	2.7	3.0	3.0	1.5	1.2	1.4	1.8	1.4	1.3	1.8	1.9	RI, SM, ^{13}C-NMR
33	γ-Muurolene	1 469	1 689	0.2	tr	0.1	0.2	0.1	0.1	0.2	0.2	0.2	0.2	0.2	0.1	RI, SM
34	Germacrene D	1 475	1 711	13.7	11.5	12.5	12.1	12.1	12.3	11.3	11.1	8.3	7.9	8.0	7.3	RI, SM, ^{13}C-NMR
35	β-Selinene	1 480	1 719	0.1	0.1	0.1	0.1	0.3	0.1	0.1	0.1	0.1	0.1	0.1	0.1	RI, SM, ^{13}C-NMR
36	4-*epi*-Cubebol	1 486	1 886	0.1	0.1	0.1	0.1	0.2	0.1	0.2	0.1	0.2	0.1	tr	tr	RI, SM
37	Bicyclogermacrene	1 489	1 733	0.4	0.3	0.3	0.6	0.1	0.1	0.3	0.2	0.3	0.6	0.7	0.2	RI, SM, ^{13}C-NMR
38	α-Muurolene	1 491	1 724	0.2	0.1	0.1	0.2	0.1	0.1	0.2	0.2	0.2	0.2	0.2	0.1	RI, SM
39	(*E,E*)-α-Farnesene	1 494	1 751	0.3	0.1	0.2	0.2	0.2	0.1	0.4	0.3	0.6	0.5	0.5	0.1	RI, SM, ^{13}C-NMR
40	β-Bisabolene	1 499	1 729	0.1	0.1	0.2	0.2	tr	0.2	0.5	0.1	0.3	0.3	0.4	0.1	RI, SM, ^{13}C-NMR
41	γ-Cadinene	1 504	1 758	0.3	0.4	0.3	0.3	0.4	1.1	1.7	0.7	0.2	0.4	0.3	0.2	RI, SM, ^{13}C-NMR
42	δ-Cadinene	1 513	1 758	0.5	0.8	0.6	0.6	1.0	0.8	0.7	0.6	1.4	1.0	1.1	1.4	RI, SM, ^{13}C-NMR
43	β-Elemol	1 533	2 079	tr	tr	tr	0.5	0.1	0.1	0.1	0.2	0.1	tr	0.1	tr	RI, SM, ^{13}C-NMR
44	(*E*)-Nerolidol	1 546	2 042	0.1	0.2	0.1	0.4	0.2	0.1	0.2	0.1	0.1	0.1	0.1	0.1	RI, SM
45	Germacrene B#	1 549	1 827	0.9	0.5	0.7	1.0	0.3	0.1	0.1	0.2	0.3	0.3	0.3	0.6	RI, SM, ^{13}C-NMR
46	Spathulenol	1 562	2 121	0.1	0.1	0.1	0.2	0.1	0.1	0.1	0.1	0.1	0.1	0.1	tr	RI, SM
47	Caryophyllene oxide	1 569	1 979	0.3	0.4	0.6	0.3	0.3	0.1	0.2	0.3	0.7	0.8	0.6	0.5	RI, SM, ^{13}C-NMR
48	Humulene oxide II	1 591	2 035	0.1	0.3	0.2	0.1	0.1	0.1	0.1	0.1	tr	tr	0.1	0.2	RI, SM, ^{13}C-NMR
49	*epi*-Cubenol	1 606	2 048	0.1	tr	0.1	0.3	tr	0.1	0.3	0.1	tr	0.1	0.1	0.1	RI, SM
50	Alismol	1 609	2 253	0.1	0.1	0.1	tr	0.1	0.1	0.1	0.2	tr	tr	-	0.1	RI, SM
51	1,10-*diepi*-Cubenol	1 616	2 055	0.1	-	tr	0.1	0.2	0.2	0.1	0.1	0.1	tr	tr	-	RI, SM
52	τ-Cadinol	1 625	2 168	-	-	-	0.2	tr	0.1	-	0.1	-	-	-	-	RI, SM, ^{13}C-NMR
53	τ-Muurolol	1 626	2 182	tr	tr	0.1	0.2	0.1	0.1	tr	0.1	0.1	-	tr	0.1	RI, SM

54	β-Himachalol	1 633	2 216	0.1	0.6	0.8	0.5	tr	0.2	0.1	tr	-	0.2	tr	tr	RI, SM, ^{13}C-NMR
55	α-Cadinol	1 636	2 227	0.1	0.1	tr	0.3	tr	0.1	0.2	0.1	0.1	-	-	tr	RI, SM, ^{13}C-NMR
56	Cadina-1(10),4-dien-8β-ol	1 674	2 283	-	-	-	-	0.1	0.1	-	0.1	-	-	-	-	RI, SM, ^{13}C-NMR
57	Benzyl benzoate	1 721	2 620	tr	tr	0.1	tr	tr	tr	tr	tr	tr	0.1	tr	tr	RI, SM, ^{13}C-NMR
58	(*E*)-Phytol	2 096	2 610	0.1	0.2	0.2	0.3	0.4	0.2	0.6	0.3	0.4	0.8	0.4	0.2	RI, SM, ^{13}C-NMR
	Hydrocarbon monoterpenes			68.0	71.9	69.8	68.3	68.8	67.3	66.0	68.4	79.0	79.7	78.9	80.4	
	Oxygenated monoterpenes			0.6	1.0	1.0	0.3	6.8	8.8	7.9	7.4	0.5	0.8	0.6	1.6	
	Hydrocarbon sesquiterpenes			26.8	24.1	25.0	27.0	21.7	21.0	22.8	21.2	17.3	16.2	17.3	15.5	
	Oxygenated sesquiterpènes			1.2	1.9	2.2	3.2	1.5	1.6	1.7	1.7	1.5	1.4	1.1	1.1	
	Other compounds			0.1	0.2	0.3	0.4	0.5	0.2	0.8	0.4	0.6	1.1	0.7	0.2	
	**Total identified**			96.7	99.1	98.3	99.2	99.3	98.9	99.2	99.1	98.9	99.2	98.6	98.8	

[a]Order of elution and percentages are given on a non-polar column (BP-1), except components with an asterisk (*), where percentages are taken on a polar column (BP-20). (#) Thermolabile compound, percentage evaluated by a combination of GC-FID and ^{13}C-NMR data. RIa, RIp: retention indices measured on non-polar and polar capillary column, respectively. (-): not detected; tr: traces level (<0.05%).

Table S3: Kaiser-Meyer-Olkin (KMO) criterion values.

Variables	Values
α-Thujene	0.797
α-Pinene	1.000
Sabinene	0.870
β-Pinene	0.998
Myrcene	0.463
β-Phellandrene	0.417
Limonene	0.973
(E)-β-Ocimene	0.499
Terpinen-4-ol	0.792
Thymol	0.944
β-Elemene	0.976
(E)-β-Caryophyllene	0.999
Valerena-4,7(11)-diene	0.479
γ-Elemene	0.866
α-Humulene	0.994
Germacrene D	0.999
γ-Cadinene	0.194
δ-Cadinene	0.289
Germacrene B	0.976
Caryophyllene oxide	0.444
Benzyl benzoate	0.924
(E)-Phytol	0.937
KMO	0.998

Table S4: Principal components eigenvalues and variances (PCA).

Principal Components	Eigenvalues	Variance (%)	Cumulative variance (%)
F1	213.138	90.711	90.711
F2	12.380	5.269	95.980
F3	4.297	1.829	97.808
F4	1.690	0.719	98.528
F5	1.025	0.436	98.964
F6	0.708	0.301	99.265
F7	0.519	0.221	99.486
F8	0.355	0.151	99.637
F9	0.216	0.092	99.729
F10	0.178	0.076	99.805
F11	0.123	0.052	99.857
F12	0.096	0.041	99.898
F13	0.056	0.024	99.922
F14	0.047	0.020	99.942
F15	0.044	0.019	99.961
F16	0.030	0.013	99.974
F17	0.022	0.009	99.983
F18	0.013	0.006	99.989
F19	0.009	0.004	99.992
F20	0.008	0.004	99.996
F21	0.005	0.002	99.998
F22	0.004	0.002	100.000

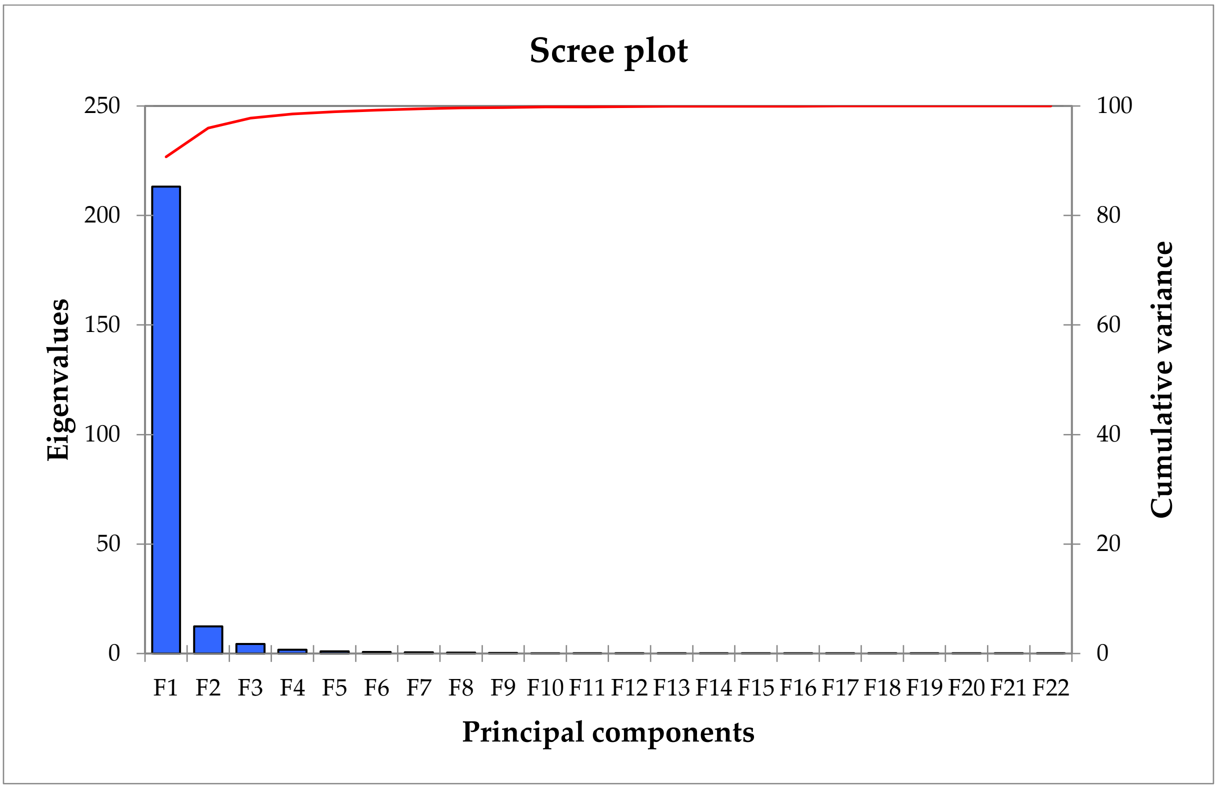

Figure S1: Scree plot of the principal components (PCA).

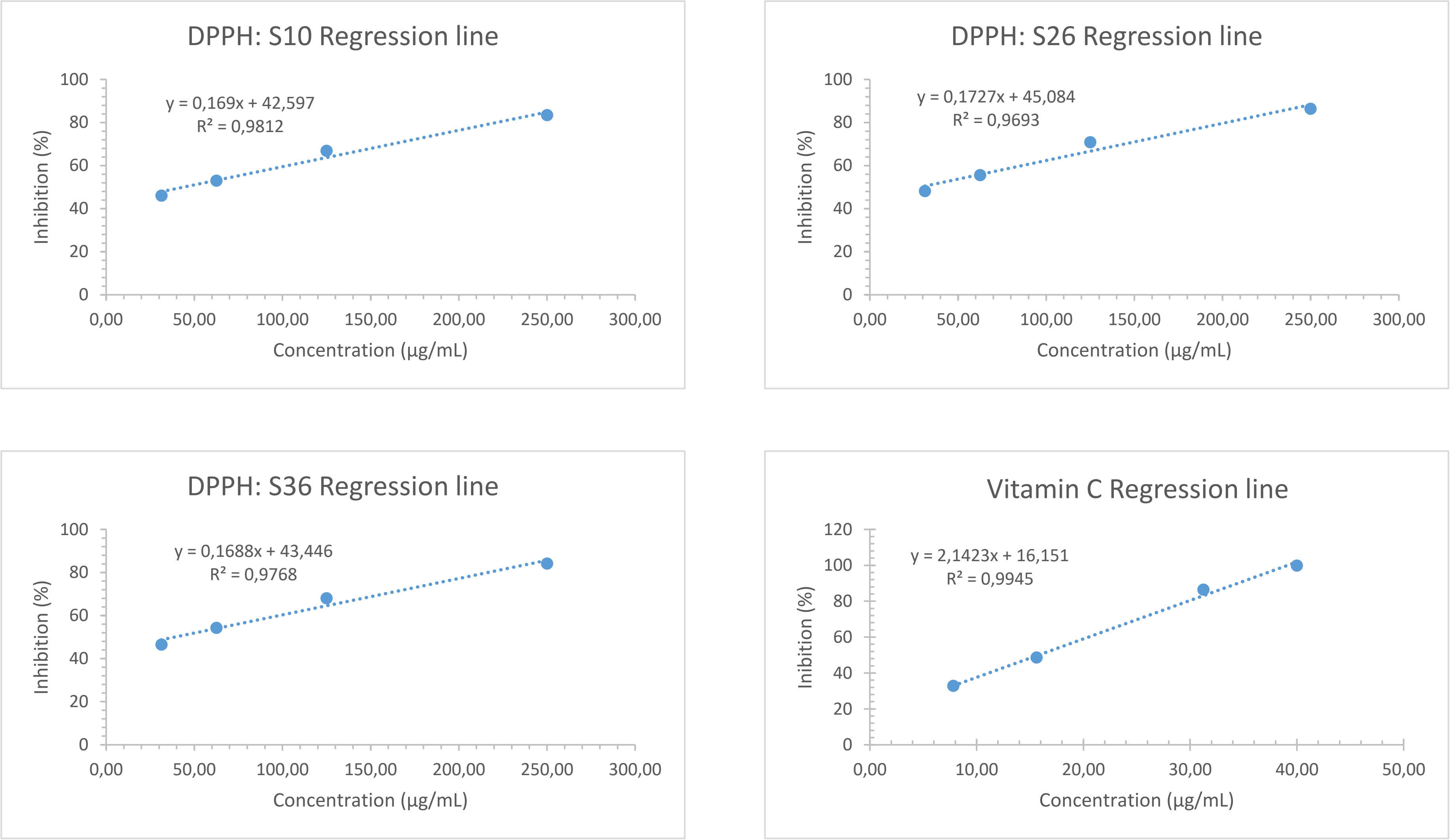

Figure S2: Linear regressions of the DDPH antioxidant assay.

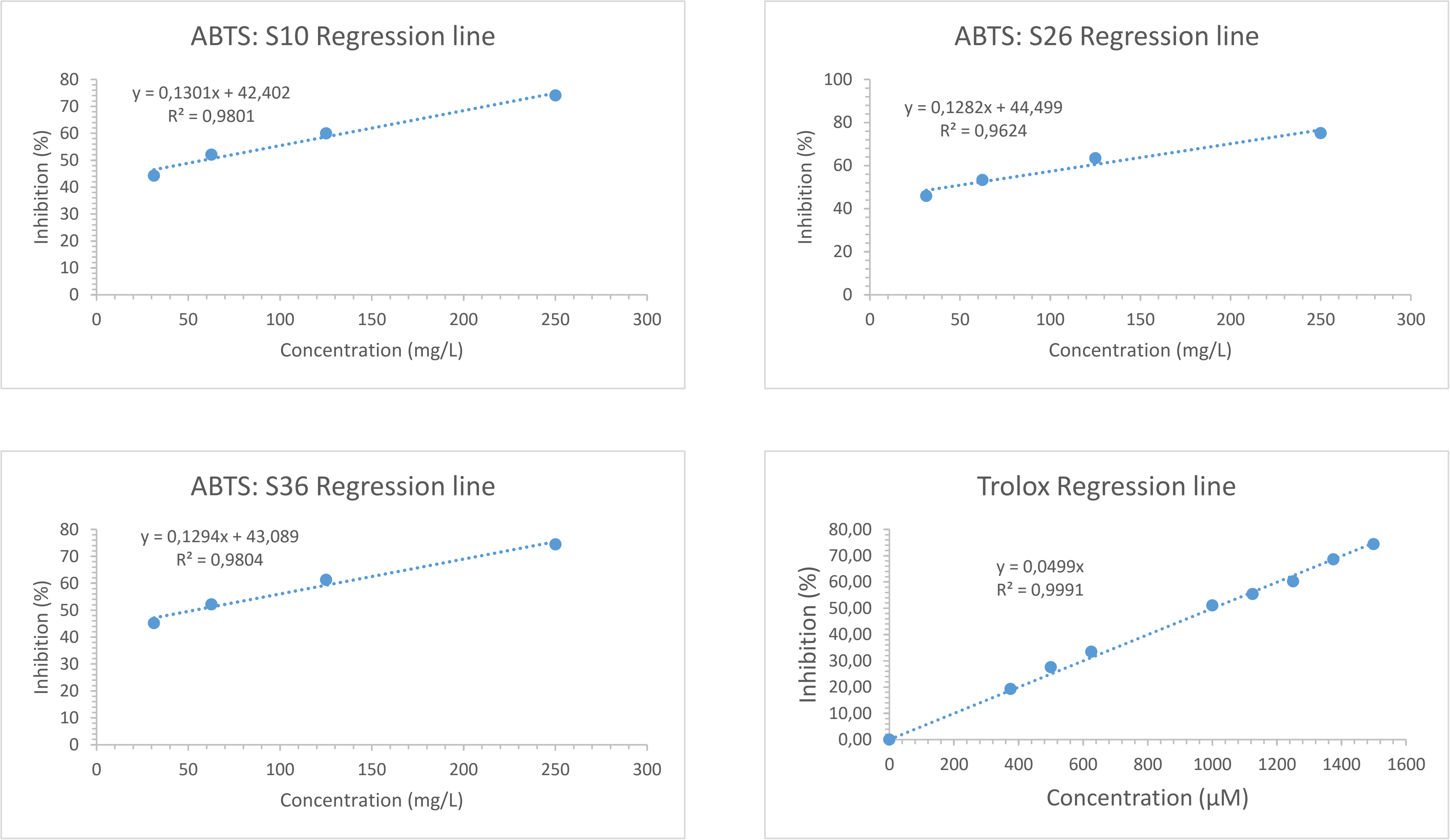

Figure S3: Linear regressions of the ABTS antioxidant assay.

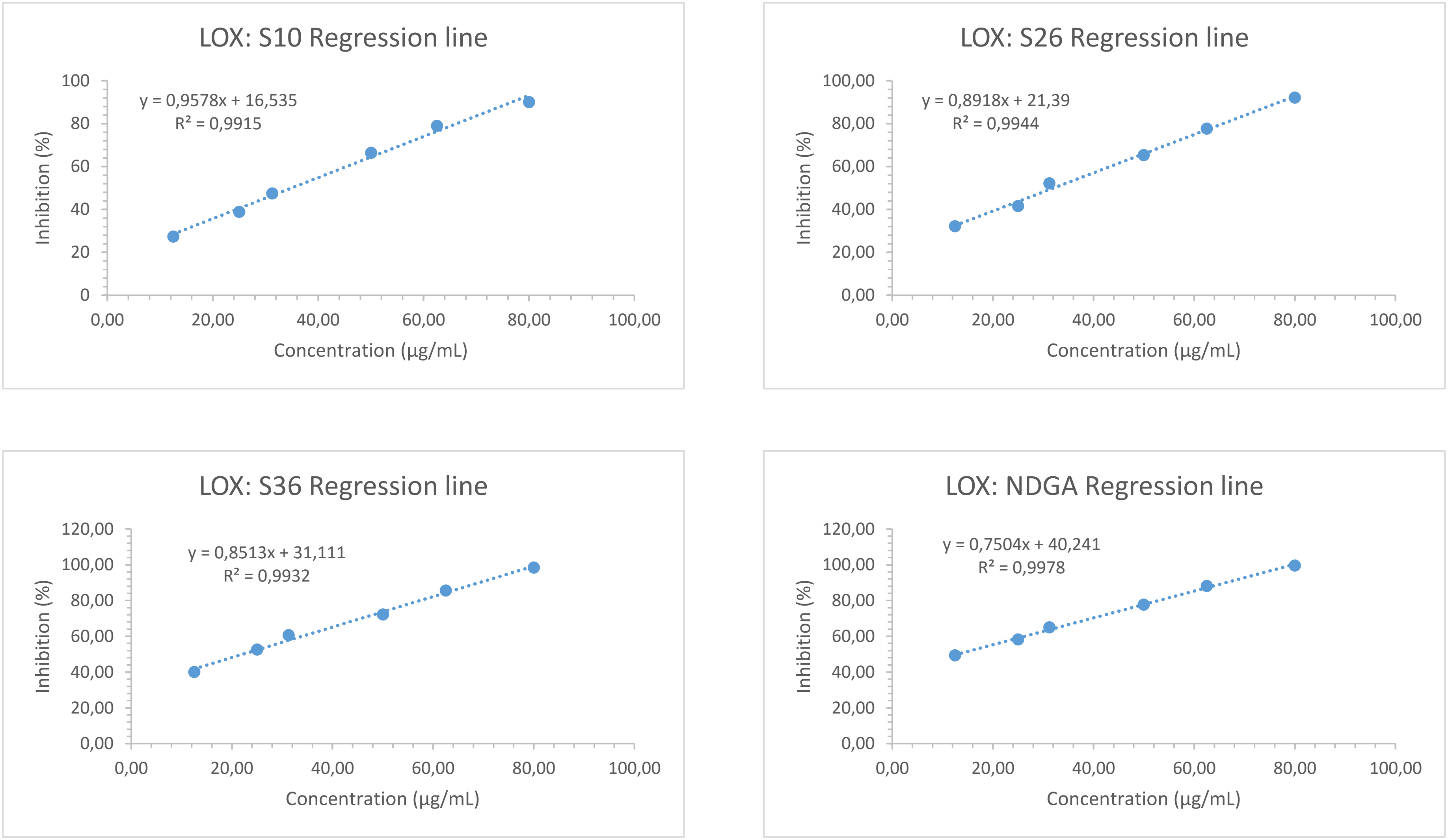

Figure S4: Linear regressions of the LOX inhibition assay.
